# Draft genome sequence of *Pseudomonas extremaustralis* strain USBA-GBX 515 isolated from Superparamo soil samples in Colombian Andes

**DOI:** 10.1186/s40793-017-0292-9

**Published:** 2017-12-15

**Authors:** Gina López, Carolina Diaz-Cárdenas, Nicole Shapiro, Tanja Woyke, Nikos C. Kyrpides, J. David Alzate, Laura N. González, Silvia Restrepo, Sandra Baena

**Affiliations:** 1 Unidad de Saneamiento y Biotecnología Ambiental (USBA), Departamento de Biología, Pontificia Universidad Javeriana, POB 56710, Bogotá, DC Colombia; 2 Department of Energy Joint Genome Institute, Joint Genome Institute, Walnut Creek, CA 94598 USA; 3 Biological Sciences Department, Universidad de los Andes, Cra 1 No. 18A – 12, Bogotá, DC Colombia

**Keywords:** *Pseudomonas extremaustralis*, *Gammaproteobacteria*, Superparamo ecosystems, Psychrophilic soils, 16S rRNA

## Abstract

Here we present the physiological features of *Pseudomonas extremaustralis* strain USBA-GBX-515 (**CMPUJU 515**), isolated from soils in Superparamo ecosystems, > 4000 m.a.s.l, in the northern Andes of South America, as well as the thorough analysis of the draft genome. Strain USBA-GBX-515 is a Gram-negative rod shaped bacterium of 1.0–3.0 μm × 0.5–1 μm, motile and unable to form spores, it grows aerobically and cells show one single flagellum. Several genetic indices, the phylogenetic analysis of the 16S rRNA gene sequence and the phenotypic characterization confirmed that USBA-GBX-515 is a member of *Pseudomonas* genus and, the similarity of the 16S rDNA sequence was 100% with *P. extremaustralis* strain CT14–3^T^. The draft genome of *P. extremaustralis* strain USBA-GBX-515 consisted of 6,143,638 Mb with a G + C content of 60.9 mol%. A total of 5665 genes were predicted and of those, 5544 were protein coding genes and 121 were RNA genes. The distribution of genes into COG functional categories showed that most genes were classified in the category of amino acid transport and metabolism (10.5%) followed by transcription (8.4%) and signal transduction mechanisms (7.3%). We performed experimental analyses of the lipolytic activity and results showed activity mainly on short chain fatty acids. The genome analysis demonstrated the existence of two genes, *lip515A* and *est515A,* related to a triacylglycerol lipase and carboxylesterase, respectively. Ammonification genes were also observed, mainly nitrate reductase genes. Genes related with synthesis of poly-hydroxyalkanoates (PHAs), especially poly-hydroxybutyrates (PHBs), were detected. The *phaABC* and *phbABC* operons also appeared complete in the genome. *P. extremaustralis* strain USBA-GBX-515 conserves the same gene organization of the type strain CT14–3^T^. We also thoroughly analyzed the potential for production of secondary metabolites finding close to 400 genes in 32 biosynthetic gene clusters involved in their production.

## Introduction

The genus 10.1601/nm.2552 *,* subclass 10.1601/nm.2068 *,* is an ubiquitous and metabolically versatile bacterial genera and is currently the genus of Gram-negative bacteria with the largest number of species [1]. Since it first description in 1894 [2], an increasing number of species has been described in diverse environments [3–5]; and now this genus comprises 255 validly named species, and 13 subspecies, according to the list published in the Namesforlife Database [6]. Psychrophilic environments are the common habitats of the 10.1601/nm.2552 genus. There are several isolated pseudomonads bacteria from water, freshwater and soils at low temperatures, such as psychrophilic strains of 10.1601/nm.2553 *,* 10.1601/nm.2606 *,* 10.1601/nm.2674 *,* 10.1601/nm.10982 *,* 10.1601/nm.8793 *,* 10.1601/nm.8795 and 10.1601/nm.8796 [5, 7–9] and recently 10.1601/nm.17787 [10]. The type strain of the species 10.1601/nm.17787 was isolated from a temporary pond in Antarctica [10, 11]. This species presents high levels of oxidative stress and cold resistance along with production of high levels of polyhydroxybutyrate (PHB) [11–13]. It is also able to tolerate and to degrade hydrocarbons, allowing it to be used in extreme environments for hydrocarbon bioremediation [14]. The polyhydroxyalkanoate synthase genes are located within a genomic island, which was probably acquired by horizontal gene transfer [11, 12, 15]. Furthermore, 10.1601/nm.17787 grows under microaerophilic conditions and forms well developed biofilms that degrades long-chain and branched alkanes, while only medium-chain length alkanes are degraded by planktonic cells [14–16].

The type strain CT14–3^T^ (10.1601/strainfinder?urlappend=%3Fid%3DDSM+17835) of 10.1601/nm.17787, and its natural derivative, the strain 14–3b (10.1601/strainfinder?urlappend=%3Fid%3DDSM+25547) have been studied for a long time, but no other strains of this species have been reported to our knowledge. We have been involved on microbial diversity studies in the Nevados National Natural Park (Nevados NNP) that harbors different extreme environments, such as permanent snows, superparamo, paramo and thermal springs associated to volcanic activity [17, 18]. These studies aim to isolate and analyze culture collections of different microbes present in these habitats.

Here we present the physiological features of 10.1601/nm.17787 strain USBA-GBX-515, isolated from soils in Superparamo ecosystems, in the northern Andes of South America, as well as its draft genome. A genomic comparison with the type strain is also presented.

## Organism information

### Classification and features

Samples were collected in 2010 from Superparamo soil samples within the Nevados NNP at >4000 m.a.s.l with soil temperature of 9.8 °C, and pH of 5.2. Paramo and superparamo are Andean ecosystems in the neotropical high mountain biome [19].

Enrichment was initiated by resuspending 10 g of rhizospheric soil samples into M9 basal medium (BM) during 30 m at 150 r.p.m. Then, the cultures were serially diluted, inoculated into M9 BM (10^−2^ to 10^−6^) and amended with 10 mM tributyrin at pH 6.0, and then incubated at 30 °C for two weeks. The M9 basal medium contained: 0.5 g NaCl, 3 g KH_2_PO_4_, 6 g Na_2_HPO_4_, 1.0 g NH_4_Cl, and 0.05 g yeast extract. We obtained pure colonies using agar plates with the same medium. Several of the pure isolates obtained were morphologically similar and 16S rRNA genes were 99% similar among them (data not shown). One strain, designated strain USBA-GBX-515, was selected for this study. The isolated bacterium was stored since the collection date at the Collection of Microorganisms of Pontificia Universidad Javeriana as 10.1601/nm.17787 strain **CMPUJU 515** (CMPUJ, WDCM857). The general features of the strain are reported in Table 1.

**Table 1:** Classification and general features of *Pseudomonas extremaustralis* strain USBA-GBX 505, according to MIGS standards [24]

MIGS ID	Property	Term	Evidence code^a^
	Current classification	Domain: Bacteria	TAS [63]
		Phylum: Proteobacteria	TAS [64]
		Class: Gammaproteobacteria	TAS [65]
		Order: Pseudomonadales	TAS [66]
		Family: Pseudomonadaceae	TAS [67]
		Genus: *Pseudomonas*	TAS [68]
		Species: *Pseudomonas extremaustralis* Type strain: CT14–3^T^	TAS [10]
	Gram-stain	Negative	IDA
	Cell shape	rod-shaped	IDA
	Motility	motile	IDA
	Sporulation	Negative	IDA
	Temperature range	4 °C – 35 °C	IDA
	Optimum temperature	30 °C	IDA
	pH range; Optimum	4.5–8.5; 7.0	IDA
	Carbon source	Hexoses	IDA
	Energy source	heterotroph	IDA
MIGS 6	Habitat	Super Paramo soil	IDA
MIGS 22	Oxygen requirement	aerobe	IDA
MIGS 15	Biotic relationship	free-living	IDA
MIGS 14	Pathogenicity		IDA
	Biosafety level	unknown	IDA
MIGS 4	Geographic location	La Olleta – Los Nevados National Natural Park	IDA
MIGS 5	Sample collection	2010	IDA
MIGS 4.1	Latitude	04 58 20 N	IDA
MIGS 4.2	Longitude	75 21 17 W	IDA
MIGS-4.4	Altitude	>4000	IDA

^a^Evidence codes: *IDA* inferred from direct assay (first time in publication); *TAS* traceable author statement (i.e., a direct report exists in the literature); *NAS* non-traceable author statement (i.e., not directly observed for the living, isolated sample, but based on a generally accepted property for the species, or anecdotal evidence). These codes are from the Gene Ontology project [69]

**Fig. 1:**
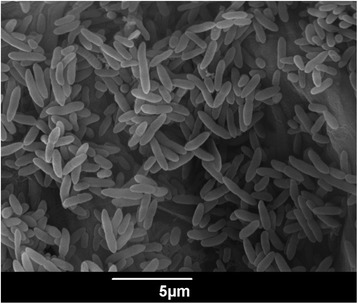
Scanning electron micrograph of *P. extremaustralis* USBA-GBX-515 in exponential phase. The image was obtained under a JSM6490 Scanning Electron Microscope at an operating voltage of 20.0 kV, using a modified protocol of Read & Jeffree [70]. Scale bar represents 5 μm

Growth was assayed at different pHs (4.5 to 8.5) following the protocols described by Rubiano et al. (2013) [20], with the optimal growth pH being 7.0. Also, different growth temperatures (from 4 °C to 35 °C) were tested and although growth was observed at all temperatures, the optimum temperature was determined as 30 °C. Strain USBA-GBX-515 is a Gram-negative rod shaped bacterium of 1.0–3.0 μm × 0.5–1 μm (Fig. 1), aerobic, motile and unable to form spores. Cells present one single flagellum. Colonies are small, smooth, circular and they did not show pigments on Luria Bertani (LB) medium but fluorescent pigments were observed on Centrimide and King B agar. Using the API ZYM strip (BioMérieuxMarcy l’Etoile, France) positive reactions were observed for catalase and oxidase. The API50CH and API 20 (BioMérieux) tests showed positive reactions for L-arginine, sodium citrate and nitrate, nitrite, and negative for starch, casein, urea, indole, D-mannitol, L-arabinose and gelatin. Strain USBA-GBX-515 exhibited alkaline phosphatase and phosphohydrolase activities. This strain presents susceptibility to imipenem, piperacilin, ticarcilin, meropenem, levofloxacin, ceftriaxone, cefoxitin and ceftazidime. On the other hand, strain USBA-GBX-515^T^ showed resistance to penicillin, colistin or polymyxin E, and nitrofurantoin.

Due to our particular interest on lipase enzymes, we also evaluated the lipolytic activity of strain USBA-GBX-515, following the protocols described in [21]. We observed growth on Tween 80, olive oil, triolein, tricaprylin and tributyrin when these compounds were used as carbon sources. We measured the lipolytic activity using *p-*nitrophenyl butyrate during its growth for 42 h at 30 °C, using tributyrin as carbon source. We detected the maximum activity, 2.0 UL μmol/L/min at 15 h at the end of the exponential phase, as previously reported for the species of the genus [22, 23]. Additionally, we observed the higher activity in the extracellular fraction than in the intracellular fraction.

**Fig. 2:**
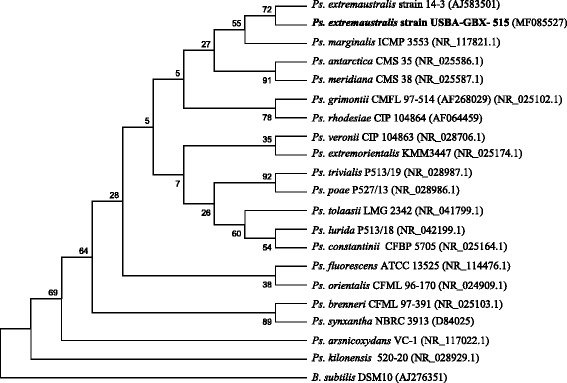
Phylogenetic tree based on 16S rRNA gene sequences showing the phylogenetic position of Pseudomonas extremaustralis USBA-GBX-515. Bootstrap values were based on 1000 resamplings. Sequence accession numbers are given in parentheses

Analysis for initial phylogenetic inferences was done using universal amplification primers 27F (5′ CAGAGTTTGATCCTGGCTCAG 3′) and 1492R (5′ TACGGYTACCTTGTTACGACTT 3′). PCR products were sequenced using Sanger technology with an eight capillary Applied Biosystems GA-3500 sequencer. Neighbor-joining phylogenetic tree reconstruction was done using MEGA 7.0.25. Phylogenetic analysis of the 16S rRNA gene sequence (Fig. 2) confirmed that USBA-GBX-515 is a member of 10.1601/nm.2552 genus. The most closely related strain was 10.1601/nm.17787 CT14–3^T^ and then, our isolate was assigned to 10.1601/nm.17787 *,* by comparison of the 16S rRNA sequence. Strain USBA-GBX-515^T^ exhibited a 100% 16S rRNA sequence identity (e-value = 0.0) with 10.1601/nm.17787 CT14–3^T^.

10.1601/nm.17787 strain USBA-GBX-515 was stored at the Collection of Microorganisms of Pontificia Universidad Javeriana (CMPUJ, WDCM857) (**ID CMPUJ U515**) with the ID USBA-GBX 515, growing aerobically on the same medium as mentioned above. Cells were preserved at −20 °C in BM supplemented with 20% (*v*/v) glycerol.

## Genome sequencing information

### Genome project history

The strain was selected to sequencing on the basis of its metabolic versatility and the biotechnological potential as revealed by previous studies [10–12]. This work is part of the bigger study aiming at exploring the microbial diversity in extreme environments in Colombia. More information can be found on the Genomes OnLine database under the study Gs0118134. The JGI accession number is 1,094,800 and consists of 69 scaffold. Table 2 depicts the project information and its association with MIGS version 2.0 compliance [24]. The USBA-GBX-515^T^ draft Genome has the ENA accession number FUYI01000001-FUYI01000069, and is also available through the Integrated Microbial Genomes system under the accession 2,671,180,025.

**Table 2:** Project information

MIGS ID	Property	Term
MIGS 31	Finishing quality	High-quality Draft
MIGS 28	Libraries used	Paired-end
MIGS 29	Sequencing platforms	Illumina HiSeq 2500
MIGS 31.2	Fold coverage	239.8
MIGS 30	Assemblers	ALLPATHS/Velvet
MIGS 32	Gene calling method	BLAST2GO
	Locus Tag	BCL77
	Genbank ID	FUYI01000069.1
	GenBank Date of Release	04–04-2017
	JGI ID	1,094,763
	JGI Date of Release	02–03-2017
	BIOPROJECT	PRJNA330486
	IMG Taxon ID	2,671,180,025
MIGS 13	Source Material Identifier	USBA 515
	Project relevance	Metabolic versatility, natural products discovery

### Growth conditions and genomic DNA preparation

10.1601/nm.17787 strain USBA-GBX-515 grew aerobically on LB medium at 30 °C. A 1 mL of overnight culture was centrifuged for 2 min at 13000 *g*. the pellet was immediately used for DNA extraction using the Wizard SV GEnomic DNA purification kit (Promega, USA). The integrity and quality of the DNA was verified using agarose gels (Sigma-Aldrich, St. Louis, USA) 0.8% (*w*/*v*) and using the NanoDropTM system (Thermo Scientific). The genomic DNA concentration was measured by the Qubit® dsDNA by fluorometric quantitation (Invitrogen, USA).

### Genome sequencing and assembly

Genomic DNA for 10.1601/nm.17787 strain USBA-GBX-515 was sequenced on a HiSeq 2500 sequencer (Illumina, SanDiego, CA, USA) with a paired-end strategy (PE150) of 300-bp reads. The sequencing platform generated 10,817,988 reads. After trimming a total of 10,000,000 paired end reads were obtained and assembled into 77 contigs and 69 scaffolds using ALLPATHS [25] and Velvet [26] softwares. All samples were processed using BUSCO [27], which offers a measure for quantitative assessment of genome assembly and annotation quality based on evolutionarily informed expectations of gene content. With the raw data (FastQ read files), the estimated genome size was calculated using different k-mer sizes in Kmergenie [28]. Finally, to obtain assembly metrics of the different genomes, QUAST [29] was run. The draft genome of 10.1601/nm.17787 strain USBA-GBX-515 consisted of 6,143,638 Mb with a G + C content of 60.9% mol. Table 3 contains all the genome statistics.

**Table 3:** Genome statistics

Attribute	Value	% of Total^a^
Genome size (bp)	6,143,638	100
DNA coding (bp)	5,503,417	89.6
DNA G + C (bp)	3,739,670	60.9
DNA scaffolds	69	100
Total genes	5665	100
Protein coding genes	5544	97.86
RNA genes	121	2.14
Pseudo genes	60	1.06
Genes in internal clusters	1731	30.56
Genes with function prediction	4524	79.86
Genes assigned to COGs	4091	72.22
Genes with Pfam domains	7255	84.70
Genes with signal peptides	608	10.73
Genes with transmembrane helices	1291	22.79
CRISPR repeats	0	

^a^The total is based on either the size of the genome in base pairs or the total number of protein coding genes in the annotated genome

### Genome annotation

Genes were identified using Prodigal [30] as part of the DOE-JGI Annotation pipeline [31, 32]. The predicted CDSs were translated and used to search the National Center for Biotechnology Information (NCBI) non-redundant database, UniProt, TIGRFam, Pfam, PRIAM, KEGG, COG, and InterPro databases. Additional gene prediction analysis and functional annotation was performed within the Integrated Microbial Genomes (IMG-ER) [33].

Biosynthetic clusters were predicted running antiSMASH [34], BAGEL3 [35] and NaPDoS [36]. AntiSMASH was run using the GenBank file generated during annotation from the IMG-ER as the input. Before running the antiSMASH server tool, ClusterFinder algorithm [37], whole-genome PFAM analysis [38] and Enzyme Commission (EC) number prediction were selected. BAGEL3 is a tool specialized in predicting RiPPs and Bacteriocins using as FASTA file as the input. Finally, the NaPDoS tool was run four times per genome; first with a FASTA nucleotide file as the input and seeking KS domains and second with the same input but seeking C domains. The third and fourth runs were with FASTA amino acid files, seeking KS and C domains respectively. A list of all biosynthetic clusters is available through IMG and IMG-ABC systems [32, 39].

### Genome properties

The genome of 10.1601/nm.17787 strain USBA-GBX-515 is 6,143,638 bp –long with a G+ C content of 60.9 mol%. A total of 5665 genes were predicted and of those, 5544 were protein coding genes and 121 RNA genes. The properties and statistics of the genome are summarized in Table 3, of the total CDSs, 72.2% represent COG functional categories. The distribution of genes into COGs functional categories is presented in Table 4. Most genes were classified in the category of amino acid transport and metabolism (10.5%), followed by transcription (8.38%) and signal transduction mechanisms (7.3%).

**Table 4:** Number of genes associated with general COG functional categories

Code	Value	% age	Description
E	511	10.95	Amino acid transport and metabolism
G	247	5.29	Carbohydrate transport and metabolism
D	38	0.81	Cell cycle control, cell division, chromosome partitioning
N	153	3.28	Cell motility
M	266	5.7	Cell wall/membrane/envelope biogenesis
B	2	0.04	Chromatin structure and dynamics
H	237	5.08	Coenzyme transport and metabolism
Z	0	0	Cytoskeleton
V	101	2.16	Defense mechanisms
C	284	6.08	Energy production and conversion
W	31	0.66	Extracellular structures
S	234	5.01	Function unknown
R	404	8.65	General function prediction only
P	282	6.04	Inorganic ion transport and metabolism
U	116	2.49	Intracellular trafficking, and secretion
I	223	4.78	Lipid transport and metabolism
X	43	0.92	Mobilome: prophages, transposons
F	106	2.27	Nucleotide transport and metabolism
O	164	3.51	Posttranslational modification, protein turnover, chaperones
A	1	0.02	RNA processing and modification
L	121	2.59	Replication, recombination and repair
Q	133	2.85	Secondary metabolites biosynthesis, transport and catabolism
T	344	7.37	Signal transduction mechanisms
K	391	8.38	Transcription
J	236	5.06	Translation, ribosomal structure and biogenesis
	1554	27,78	Not in COG

### Insights from the genome sequence

**Fig. 3:**
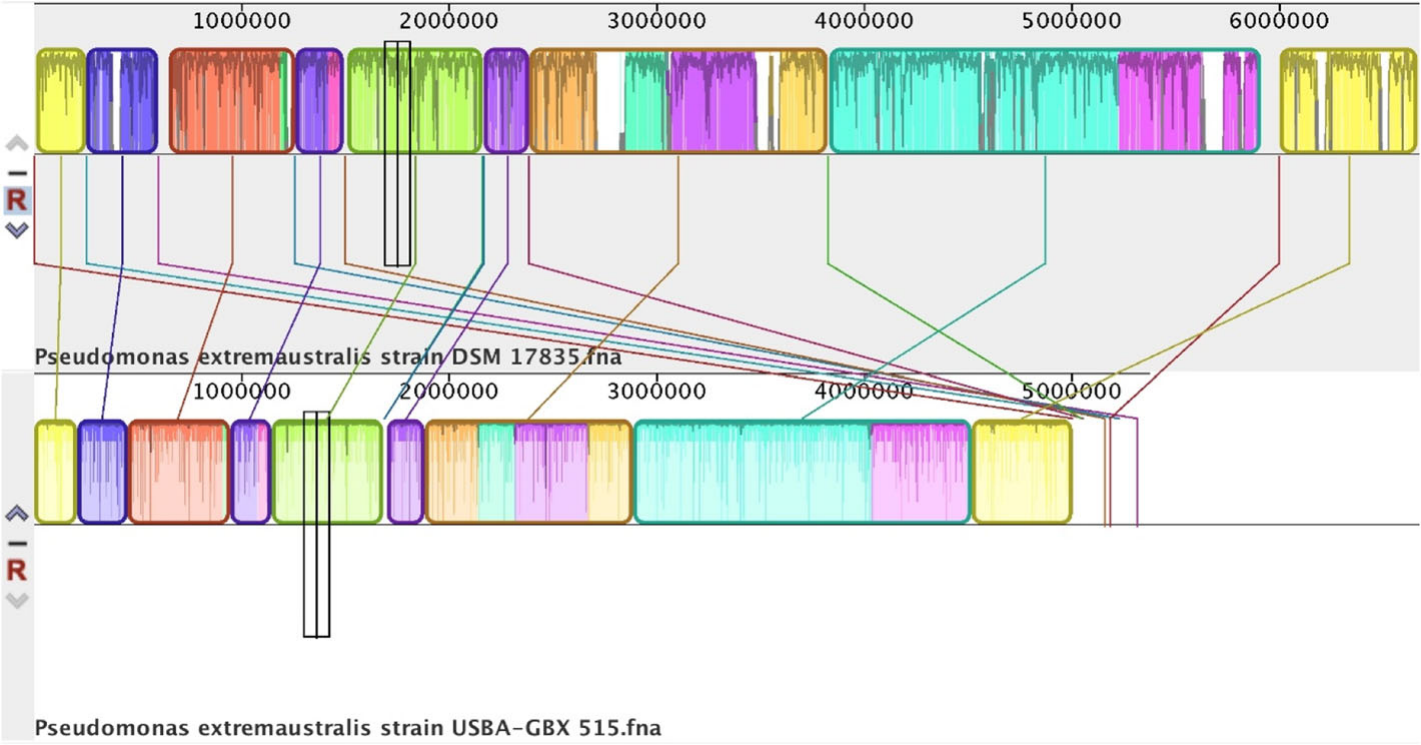
Multiple Alignment performed using Mauve [42] of *P. extremaustralis* genomes. The type strain CT14–3^T^ of *Pseudomonas extremaustralis* (17835^T^) is shown in the top and the strain USBA-GBX 515 described here at the botton. Conserved blocks are represented with direct lines from type strain to our strain showing synteny of genes among the genome. Small regions between conserved blocks from type strain are assembled in small contigs at the end of our genome

We performed taxonomic genome comparisons between 10.1601/nm.2552 USBA-GBX-515 and 10.1601/nm.17787 strain CT14–3^T^. The average nucleotide identity (ANI) calculated with the MiSI (Microbial Species Identifier) method [40] is 98.9% with an Alignment Fraction (AF) of 0.91. Using GGDC web server version 2.1 [41], the DNA-DNA hybridization was calculated, and it showed 96.7% of similarity; the difference in G + C content was less than 1% (0.27 of difference) within both strains. Finally, a pairwise genome alignment performed with Mauve [42] between our strain and the type strain CT14–3^T^ of 10.1601/nm.17787 (17835^T^) was performed, showing the similarity and conserved synteny of genes (Fig. 3). There are few regions that were unassembled in our genome and those remain in small separated contigs. All analyses corroborate the affiliation of our strain to the species 10.1601/nm.17787.

This isolate was screened for lipolytic activities, and the genome analysis showed two genes *lip515A* and *est515A* related to a triacylglycerol lipase and carboxylesterase, respectively. Both genes showed a conserved α/β hydrolase motif which is common in lipolytic enzymes [43, 44], and are required for the lipids and fatty acid metabolisms. Particularly, the deduced amino acid sequence (296aa) from Gene *lip515A* showed an identity of 49% (E value 3e-80) with a triacylglycerol lipase from 10.1601/nm.2607 [45], while gene *est515A* had a 68% identity (E value 2 e-85) with a hypothetical protein from 10.1601/nm.2834 sp. TT2012.

Ammonification genes were also observed, mainly nitrate reductase genes (*narG,H,I,J,L,X and napA*). Markers of nitrifying bacteria, *norB* and *nosZ* reductases were found, both genes were described previously in 10.1601/nm.2690, a nitrate respiring bacterium [46]. We found a *norVW* gene, which has a role in protection against reactive nitrogen intermediates [47, 48]. A total of 732 genes were identified to play a role in amino acid transport and metabolism, which depends on nitrogen fixation metabolism.

The presence of proline operon *proHJ* and p*roA* gene demonstrate the response to high osmolarity due to the de novo synthesis of proline as a stress protectant of the cell [49]. Cell protection from toxic effects of hydrogen peroxide was determined by the presence of the catalase (*katE*) gene.

Similar to previously observed on 10.1601/nm.17787 CT14–3^T^, we found genes related with the synthesis of poly-hydroxyalkanoates (PHAs). Especially poly-hydroxybutyrates genes (PHBs), were detected in the bacterial genome using BlastP (e-value <0.05 and 90% coverage of the gene). The *phaABC* operon was present, containing the PHA synthase (phaC), β-ketothiolase (phaA), and NADP-dependent acetoacetyl-CoA reductase (phaB). The *phbABC* operon is also present into the genome, corresponding to the same enzymes.

**Fig. 4:**
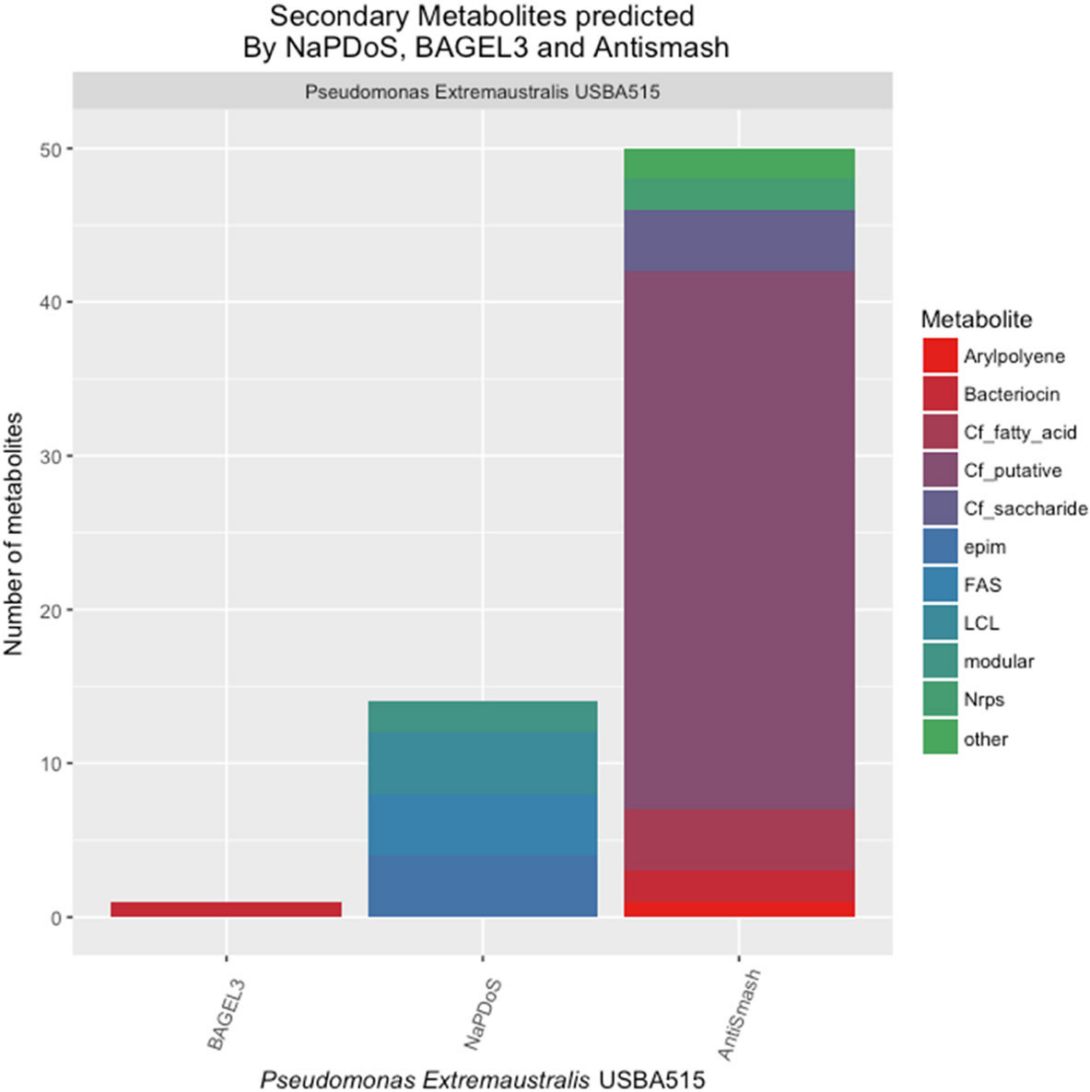
Secondary metabolites predicted by antiSMASH 3.0 [71], BAGEL3 [35] and NaPDoS [36] softwares in the genome of *Pseudomonas extremaustralis* strain USBA-GBX-515

In order to gain knowledge about the strain USBA-GBX-515^T^, we explored the potential production of secondary metabolites by data mining (Fig. 4). The genes responsible for the secondary metabolites were organized in 32 biosynthetic gene clusters using IMG tools. Those contained approximately 400 genes, the 78% of clusters were designed as putative, whilst 22% were related to NRPS and bacteriocin, but it was not possible to identify known metabolites.

**Fig. 5:**
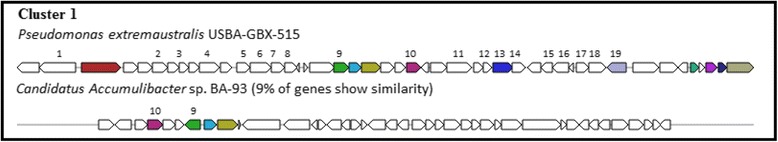
Genetic map of lipopolysaccharide biosynthetic gene cluster 1 detected by AntiSMASH 3.0. The genes were designated by colors. Same color means equal genes in different strains, not-colored means other genes. 1. pyruvate dehydrogenase subunit E1; 2. UDP-glucose:(heptosyl) LPS alpha 1,3-glucosyltransferase WaaG; 3. hypothetical protein PA5008; 4. Serine/threonine protein kinase; 5. sugar ABC transporter substrate-binding protein; 6. ABC transporter ATP-binding protein; 7. bifunctional carbohydrate binding and transport protein; 8. bifunctional carbohydrate binding and transport protein; 9. glycosyl transferase family protein; 10. glycosyl transferase family protein; 11. lipid A export permease/ATP-binding protein MsbA; 12. adenylyl-sulfate kinase; 13. bifunctional heptose 7-phosphate kinase/heptose 1-phosphate adenyltransferase; 14. Epimerase; 15. hypothetical protein PA4992;16. FAD-dependent oxidoreductase; 17. transcriptional regulator; 18. hypothetical protein PA4974; 19. 3-deoxy-D-manno-octulosonic acid transferase

**Fig. 6:**
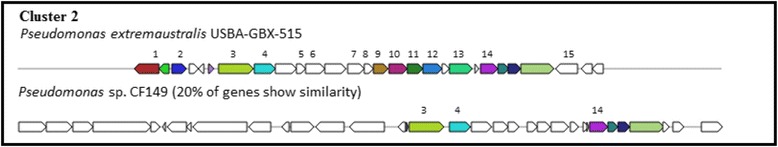
Genetic map of colanic acid biosynthetic gene cluster 2 detected by AntiSMASH 3.0. The genes were designated by colors. Same color means equal genes in different strains; not colored means other genes. 1. Thiosulfate sulfurtransferase; 2. transcriptional regulator; 3. nucleotide sugar dehydrogenase; 4. colanic acid production tyrosine-protein kinase; autokinase; Ugd phosphorylase; 5–7. hypothetical protein; 8. putative acyl transferase; 9. Glycosyl transferase; 10. GDP-D-mannose dehydratase, NAD(P)-binding; 11. bifunctional GDP-fucose synthetase: GDP-4-dehydro-6-deoxy-D-mannose epimerase/ GDP-4-dehydro-6-L-deoxygalactose reductase; 12. colanic acid biosynthesis glycosyl transferase WcaI; 13. mannose-1-phosphate guanylyltransferase/mannose-6-phosphate isomerase

**Fig. 7:**
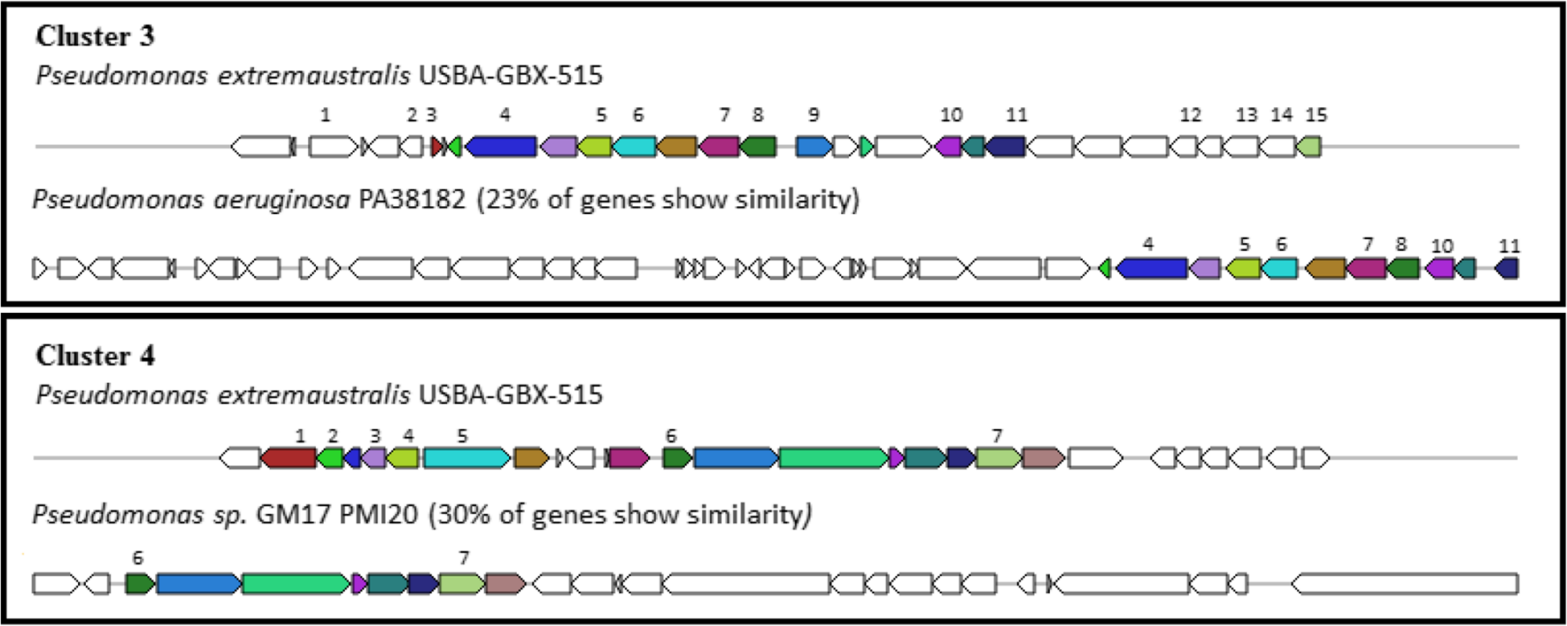
Genetic map of O-antigen biosynthetic gene cluster 3 and 4 detected by AntiSMASH 3.0. The genes were designated by colors. Same color means equal genes in different strains; not colored means other genes. Cluster 3: 1. Transporter; 2. GntR family transcriptional regulator; 3. TetR family transcriptional regulator; 4. nucleotide sugar epimerase/dehydratase WbpM; 5. NAD-dependent epimerase/dehydratase family protein; 6. glycosyltransferase WbuB; 7. PREDICTED: UDP-glucuronic acid decarboxylase 6; 8. bifunctional UDP GlcNAc C6 dehydratase/C5 epimerase PseB; 9. CPS-53 (KpLE1) prophage; bactoprenol glucosyl transferase; 10. imidazole glycerol phosphate synthase subunit HisF; 11. LPS biosynthesis; 12. 3-oxoacyl-ACP reductase; 13. hypothetical protein; 14. pilin glycosylation protein PglB; 15. mannose-1-phosphate guanyltransferase beta. Cluster 4: 1. two-component sensor; 2. NAD(P)-dependent oxidoreductase; 3. hypothetical protein spr0320; 4. dTDP-D-glucose 4,6-dehydratase; 5. ethanolamine-phosphate phospho-lyase; 6. Epimerase; 7. pellicle/biofilm biosynthesis glycosyltransferase PelF

**Fig. 8:**
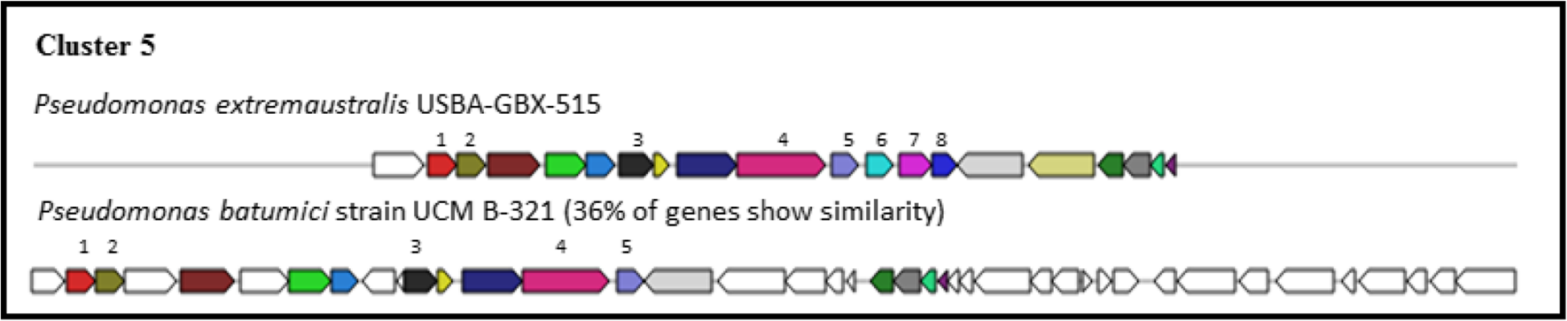
Genetic map of glidobactin biosynthetic gene cluster 5 detected by AntiSMASH 3.0. The genes were designated by colors. Same color means equal genes in different strains; not colored means other genes. 1. ABC transporter permease; 2. ABC transporter ATP-binding protein; 3. prolyl aminopeptidase; 4. glucan biosynthesis glucosyltransferase; 5. amino acid ABC transporter substrate-binding protein; 6. ABC transporter, PAAT family; 7. ABC transporter permease; 8. ABC transporter substrate-binding protein

**Fig. 9:**
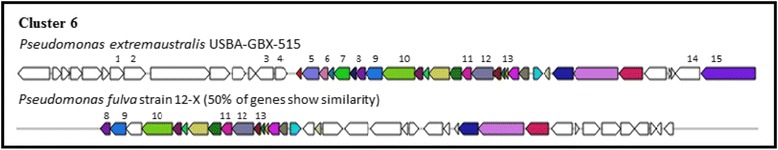
Genetic map of arylpolyene saccharide biosynthetic gene cluster 6 detected by AntiSMASH 3.0. The genes were designated by colors. Same color means equal genes in different strains, not-colored means other genes. 1. 3-dehydroquinate synthase; 2. hypothetical protein PA5037; 3. major facilitator superfamily transporter; 4. Transcriptional regulator; 5. 3-oxoacyl-(acyl-carrier-protein) synthase II FabF; 6. beta-ketoacyl synthase; 7. SAM-dependent methyltransferase type 11; 8. FAD-binding protein; 9. MMPL family efflux pump permease component. 10. glycosyl transferase; 11. AMP dependent synthase; 12. acyl carrier protein; 13. Acyltransferase; 14. acyl-CoA dehydrogenase; 15. fused DNA-binding transcriptional regulator/proline dehydrogenase/pyrroline-5-carboxylate dehydrogenase

**Fig. 10:**
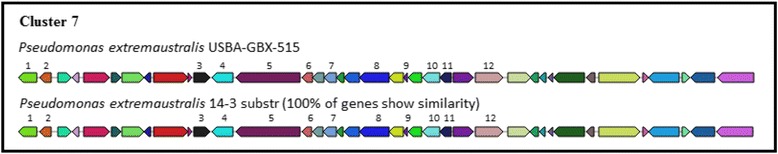
Genetic map of coronatine biosynthetic gene cluster 7 detected by AntiSMASH 3.0. The genes were designated by colors. Same color means equal genes in different strains; not colored means other genes. 1. hypothetical protein PA4617; 2. quorum-sensing control repressor; 3. Motility regulator; 4. serine hydroxymethyltransferase; 5. hypothetical protein PA4604; 6. threonine transporter RhtB; 7. Thioesterase; 8. non-ribosomal peptide synthetase; 9. coronamic acid synthetase CmaD; 10. hypothetical protein; 11. transcriptional regulator of proline and 4-hydroxyproline utilization HypR; 12.ABC transporter ATP-binding protein

**Fig. 11:**
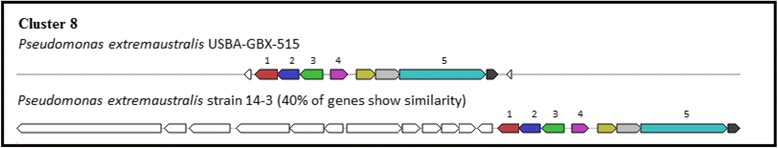
Genetic map of Mangotoxin biosynthetic gene cluster 8 detected by AntiSMASH 3.0. The genes were designated by colors. Same color means equal genes in different strains; not colored means other genes. 1. ABC transporter permease; 2. ABC transporter ATP-binding protein; 3. hypothetical protein PA2310; 4. transcriptional regulator of proline and 4-hydroxyproline utilization HypR; 5. non-ribosomal peptide synthetase

**Fig. 12:**
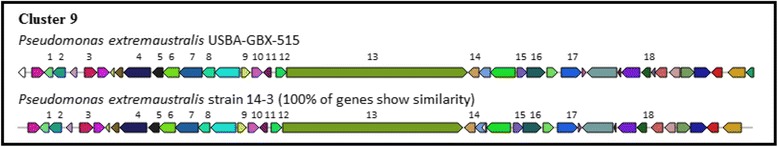
Genetic map of Pyoverdine biosynthetic gene cluster 9 detected by AntiSMASH. The genes were designated by colors. Same color means equal genes in different strains; not colored means other genes. 1. two-component system response regulator; 2. two-component system response regulator; 3. chemotaxis signal transduction system response regulator CheV; 4. geranyl-CoA carboxylase subunit alpha; 5. Isohexenylglutaconyl-Coa hydratase; 6. citronellyl-CoA dehydrogenase; 7. geranyl-CoA carboxylase subunit beta; 8. citronellol- dehydrogenase; 9. atu genes repressor; 10. amino acid ABC transporter substrate-binding protein; 11. extracytoplasmic-function sigma-70 factor; 12. pyoverdine biosynthesis protein PvdG; 13. Peptide synthase; 14. thiol:disulfide interchange protein DsbG; 15. two-component sensor histidine kinase; 16. two-component response regulator; 17. diaminobutyrate--2-oxoglutarate aminotransferase; 18. pseudouridine synthase

Using antiSMASH 3.0 platform we detected 57 cluster of biosynthetic genes. The 56% of clusters were classified as putative. Two biosynthetic clusters (classified as putative) were assigned to fengycin and alginate clusters. Fengycin is a cyclic lipopeptide acting against phytopathogenic viruses, bacteria, fungi, and nematodes. The lipopeptides are synthesized at modular multienzymatic templates [50, 51]. The polymer alginate had been identified mainly in the genus 10.1601/nm.2552 as an exopolysaccharide involved on biofilm formation and pathogenicity [52]. A total of 24.5% of the clusters were classified as saccharides. Five of the biosynthetic clusters included in this category were related to lipopolysaccharide, pseudopyronine, colonic acid, O-antigen and glidobactin. The proteins coded by the cluster associated to lipopolysaccharide (LPS) (Fig. 5) are secreted to the outer surface and the cluster is expressed as a mechanism of resistance to detergents and hydrophobic antibiotics [53]. Colanic acid is an extracellular polysaccharide related to desiccation resistance [54] and to adhesion as pathogenic factor [55] (Fig. 6). O-antigen is a lipopolysaccharide which is associated to adhesion [56] (Fig. 7). The last saccharide cluster is related to glidobactin (Fig. 8), a PKS/NPRS cytotoxic compound which is an antifungal and antitumor antibiotic complex [57]. The structure of this cluster shows a 36% similarity to the cluster of “10.1601/nm.2552 *batumici”* strain UCM B-321. A PKS/NRPS compound has been founded from “10.1601/nm.2552 *batumici”* named batumin which exhibits potent and selective antibiotic activity against *Staphylococcus* species [58]. A biosynthetic gene cluster related to arylpolyene-saccharide was detected (Fig. 9), and this metabolite has a similar structure to a pigment produced by members of the genus 10.1601/nm.2208 and 10.1601/nm.8248 *,* which is involved on Gram negative bacteria protection against exogenous oxidative stress [37]. Other clusters were associated to coronatine (Fig. 10) and mangotoxin (Fig. 11) compounds. Both are antimetabolites related to phytotoxins. The coronatine acts as a virulence factor and induces hypertrophy, inhibits root elongation, and stimulates ethylene production [59]. The mangotoxin is a small peptidic molecule, which inhibits the biosynthesis of essential amino acids, resulting in an amino acid deficiency [60]. These toxins could be used as herbicides such as glufosinate and bialaphos, two commercial herbicides that mimic bacterial toxins [60]. Finally, we found a cluster related to pyoverdine (Fig. 12), a nonribosomal peptide siderophore [61].

According to NapDOs program [36], several genes were related to fatty acid biosynthesis, particularly two genes *fat478* and *fat3803* (related to proteins FabB and FabF, respectively); those proteins are chain elongation condensing enzymes (synthases) that control fatty acid composition and influence the rate of fatty acid production [37].

Using BAGEL3 we found the cluster class III related to S-type Pyocin, a compound with a killing activity causing cell death by DNA breakdown through endonuclease activity [62].

## Conclusions

The strain USBA-GBX-515 isolated from soils associated to superparamo from Andean ecosystems, is a moderate psicrophilic and denitrifier organism. The different genetic indices, the phylogenetic analysis of the 16S rRNA gene sequence and the phenotypic characterization confirmed that USBA-GBX-515 belongs to the 10.1601/nm.17787 species. In addition, the pairwise genome alignment between our strain and the type strain CT14–3^T^ of 10.1601/nm.17787 (17835^T^) showed high similarity and conserved synteny of genes. Based on physiological characterization of this strain, we demonstrated its potential as lipolytic organism. On the other hand, based on a thorough analysis of the genome, we reported this strain as a potential producer of secondary metabolites, such as bactericin pyiocin and PK/NRPS associated to glidobactin, a potential cytotoxic compound. This strain could be also an interesting producer of secondary metabolites such as pyoverdine or glidobactin.

## Abbreviations

CMPUJU: Colección de Microorganismos de la Pontificia Universidad Javeriana
DSM: Deutsche Sammulung von Mikroorganismen
IDA: Inferred from direct assay
m.a.s.l: Meters above sea level
MIGS: Minimum information about a genome sequence
MIGS: Minimum information about a genome sequence
NAS: Non-traceable
PKS/NRPS: Polyketide synthase/non-ribosomal peptide synthetase
TAS: Traceable author statement
WDCM: World Data Center for Microorganisms
